# Validity of thermography for measuring burn wound healing potential

**DOI:** 10.1111/wrr.12786

**Published:** 2019-12-14

**Authors:** Michelle E. Carrière, Louise E. M. de Haas, Anouk Pijpe, Annebeth Meij‐de Vries, Kim L. M. Gardien, Paul P. M. van Zuijlen, Mariëlle E. H. Jaspers

**Affiliations:** ^1^ Burn Center and Department of Plastic, Reconstructive and Hand surgery Red Cross Hospital Beverwijk The Netherlands; ^2^ Department of Plastic, Reconstructive and Hand Surgery Amsterdam UMC, Vrije Universiteit Amsterdam, Amsterdam Movement Sciences Amsterdam The Netherlands; ^3^ Association of Dutch Burn Centers Beverwijk The Netherlands; ^4^ Department of Epidemiology and Biostatistics, Amsterdam UMC Vrije Universiteit Amsterdam, Amsterdam Public Health research institute Amsterdam The Netherlands

## Abstract

Accurate assessment of burn wound depth and the associated healing potential is vital in determining the need for surgical treatment in burns. Infrared thermography measures the temperature of the burn wound noninvasively, thereby providing indirect information on its blood flow. Previous research demonstrated that a small, low‐priced, handheld thermal imager has an excellent reliability, but a moderate validity for measuring burn wound healing potential. A new and more sensitive version of this convenient device has become available. The aim of this study was to evaluate the validity of thermography for measuring burn wound healing potential, compared to Laser Doppler Imaging (LDI) as a reference standard. Thermal images and LDI scans were obtained from burn wounds between 2 and 5 days postburn. Temperature differences between burned and nonburned skin (Δ*T*) were calculated. To evaluate validity, Δ*T* values were compared to the healing potential categories assessed by LDI. Two receiver operating characteristic curves were created and two Δ*T* cutoff values were calculated to illustrate the ability to discriminate between burn wounds that heal in a time period of less than 14 days, between 14 and 21 days, and more than 21 days. Between June and October 2018, 43 burn wounds in 32 patients were measured. Δ*T* cutoff values of 0.6°C (sensitivity 68%, specificity 95%) and −2.3°C (sensitivity 30%, specificity 95%) were calculated to discriminate between burn wounds that heal in <14 and ≥14 days, and burn wound that heal in ≤21 and >21 days, respectively. This study shows a good validity of the feasible thermal imager for the assessment of burn wound healing potential. Therefore, we consider it a promising technique to be used for triage in local hospitals and general practices, and as a valuable addition to clinical evaluation in burn centers.

## 1 INTRODUCTION

Accurate assessment of burn wound severity (i.e., depth and the associated healing potential) is vital in predicting the occurrence of scarring and determining the need for surgical treatment in burns. It is important to discriminate between burn wounds that heal within 14 days, which rarely cause scarring and can be treated conservatively with topical treatment, and between burn wounds that heal in a time period longer than 21 days, which often cause (problematic) scarring and require surgical treatment. Overestimation of burn wound severity can result in unnecessary surgery, while underestimation may lead to surgical delay and an increased risk of hypertrophic scarring.^1^, ^2^, ^3^, ^4^ Burn physicians estimate burn wound severity based on the patient's case history together with clinical evaluation of visual and tactile wound characteristics.^5^ Although clinical evaluation is the most frequently used method worldwide,^6^ it has been shown that its accuracy ranges between 50% and 71%, depending on the experience of the observer.^7^, ^8^, ^9^, ^10^, ^11^, ^12^ It remains difficult to visually determine the degree of tissue damage. The heterogeneity of burn wounds and the possibility of depth conversion make this even more challenging.^13^, ^14^ Therefore, a noninvasive, objective technique providing early and accurate burn wound assessment is needed to assist clinicians in their clinical judgment.

Several objective burn wound assessment methods are based on imaging skin perfusion. The extent of a burn injury is related to the amount of remaining microvascular blood flow,^15^ and therefore reflects the burn wound's healing potential. Laser Doppler Imaging (LDI) is the most well‐known and frequently used technique, which provides accurate healing potential measurements between 2 and 5 days postburn.^13^, ^16^ Another measurement technique related to skin perfusion is infrared thermography. Thermal imagers display the temperature distribution of the skin in a thermal image by detecting infrared emission from the skin. Several studies have examined the diagnostic role of different types of thermal imagers in burn wound assessment.^17^, ^18^, ^19^, ^20^, ^21^, ^22^ These studies concluded that areas of deeply burned skin appear colder on a thermal image than unaffected skin. The temperature decrease in deeply burned skin is primarily caused by the destruction of the subdermal plexus, but also the reduced metabolism in injured cells may play a role.^23^ As opposed to deep burns, superficial burns show higher temperatures than unaffected skin, which may be caused by vasodilatation, inflammation, edema, and loss of the epidermal layer.^17^, ^23^ Recently, small, low‐priced, handheld thermal imagers became available. These thermal imagers can produce easy and fast measurements attached to a mobile device or tablet.^24^, ^25^ Earlier work from our study group showed that one of these feasible thermal imagers had an excellent reliability (intraclass correlation coefficient: 0.99, standard error of measurement: 0.20°C), but a moderate validity (area under the curve of 0.69) for measuring burn wound healing potential,^25^ when compared to the observed healing time. Therefore, the aim of this study was to evaluate the validity of a newer version of the thermal imager, for measuring burn wound healing potential, compared to LDI as a reference standard.

## 2 MATERIALS AND METHODS

### 2.1 Patient selection

Between June and October 2018 consecutive patients admitted to the Burn Center or referred to the outpatient clinic of the Red Cross Hospital in Beverwijk were screened for eligibility. Dutch‐speaking patients of all ages with at least one burn wound between 2 and 5 days postburn were included. Burn wounds had to measure more than 4 cm in diameter, adjacent to an area of unaffected skin. Patients that were incompetent to give written informed consent, or had chemical burns or preexisting vascular comorbidities, such as Raynaud's disease, were excluded from participation. Patients with visible signs of infection (i.e., severe redness and/or edema) around the burn wound were also excluded. The Medical Ethics Committee of VU University Medical Centre approved the study protocol (reference number: IRB00002991). Written informed consent was obtained from all patients.

### 2.2 Thermal imager

**Figure 1:**
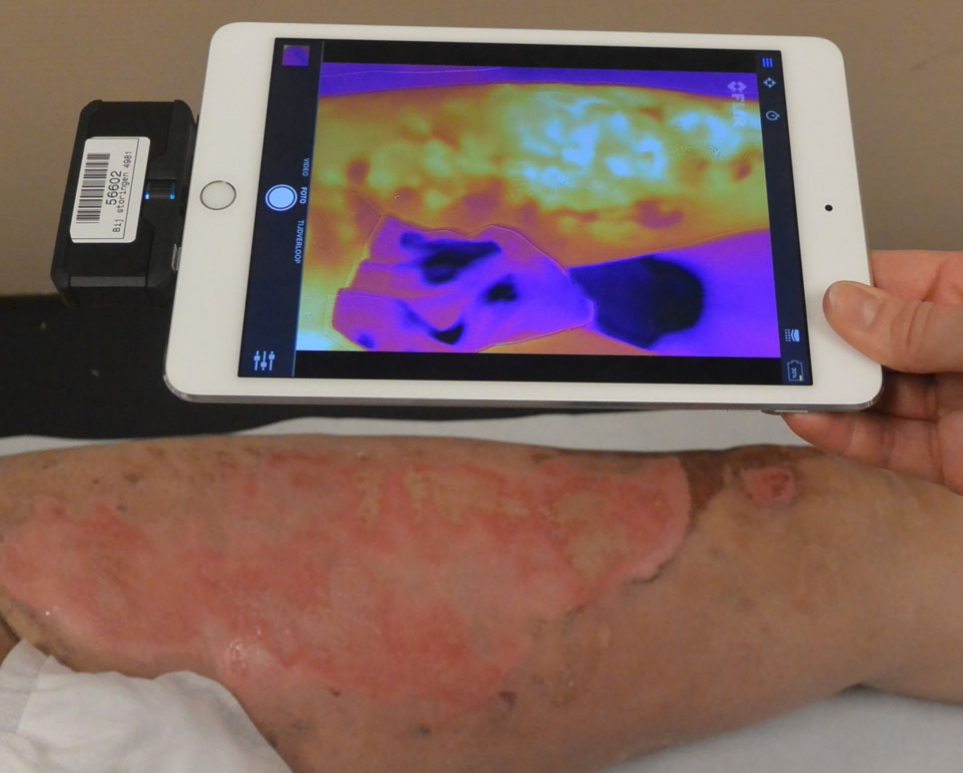
Thermography measurement with the thermal imager attached to an iPad mini [Color figure can be viewed at https://wileyonlinelibrary.com]

The thermal imager (FLIR Systems, Inc., Wilsonville, OR) was attached to an iPad mini (Apple, Inc. Cupertino, California) to produce thermal images (Figure 1). The thermal imager weighs 36.5 g and has the following dimension: 68 × 34 × 14 mm (height, width, depth). It contains two imagers, a Lepton thermal sensor (160 × 120 pixels) and a visible VGA imager (1400 × 1080 pixels). These two images are merged, resulting in one thermal image with a resolution of 1400 × 1080 pixels. The thermal imager is able to measure temperature differences as small as 0.1°C, between −20 and 400°C (https://www.flir.com/products/flir-one-pro/).

### 2.3 Laser Doppler imaging

**Figure 2:**
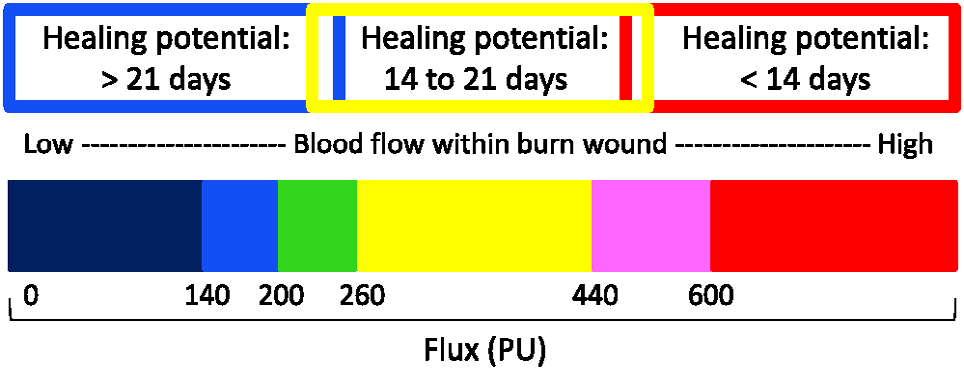
Validated color‐coded palette for LDI interpretation. *Source*: Adapted from Moor LDI2‐BI user manual [Color figure can be viewed at https://wileyonlinelibrary.com]

The MoorLDI2Burn Imager (Moor Instruments, Axminster, United Kingdom) was used as a reference standard. This device uses a low‐intensity laser beam to scan across the tissue surface of the burn wound. Moving red blood cells cause a Doppler shift of the laser, which is captured by a moving mirror. The level of perfusion (perfusion units) is visualized in a color‐coded map (Figure 2). The colors red, yellow, and blue correspond to the burn wound healing potential categories <14, 14 to 21, or >21 days, respectively.^16^ The level of perfusion in the transition between these categories is displayed by the colors green and pink.

### 2.4 Study procedure and analysis of images

Measurements were obtained with the thermal imager and the LDI by trained researchers (MC, LH) between 2 and 5 days postburn. Burn wounds were cleaned, dried, and dressing material, including ointments, as well as blisters and necrotic skin were removed if possible. Heat lamps and other external heat sources were switched off at least 10 minutes before measurements. First, the burn wound of interest and a reference area of healthy skin were captured in the same thermal image. Taking the zone of hyperemia into account, the reference area was chosen at least 3 cm next to the burn wound. Next, a LDI scan of the same burn wound was acquired.

**Figure 3:**
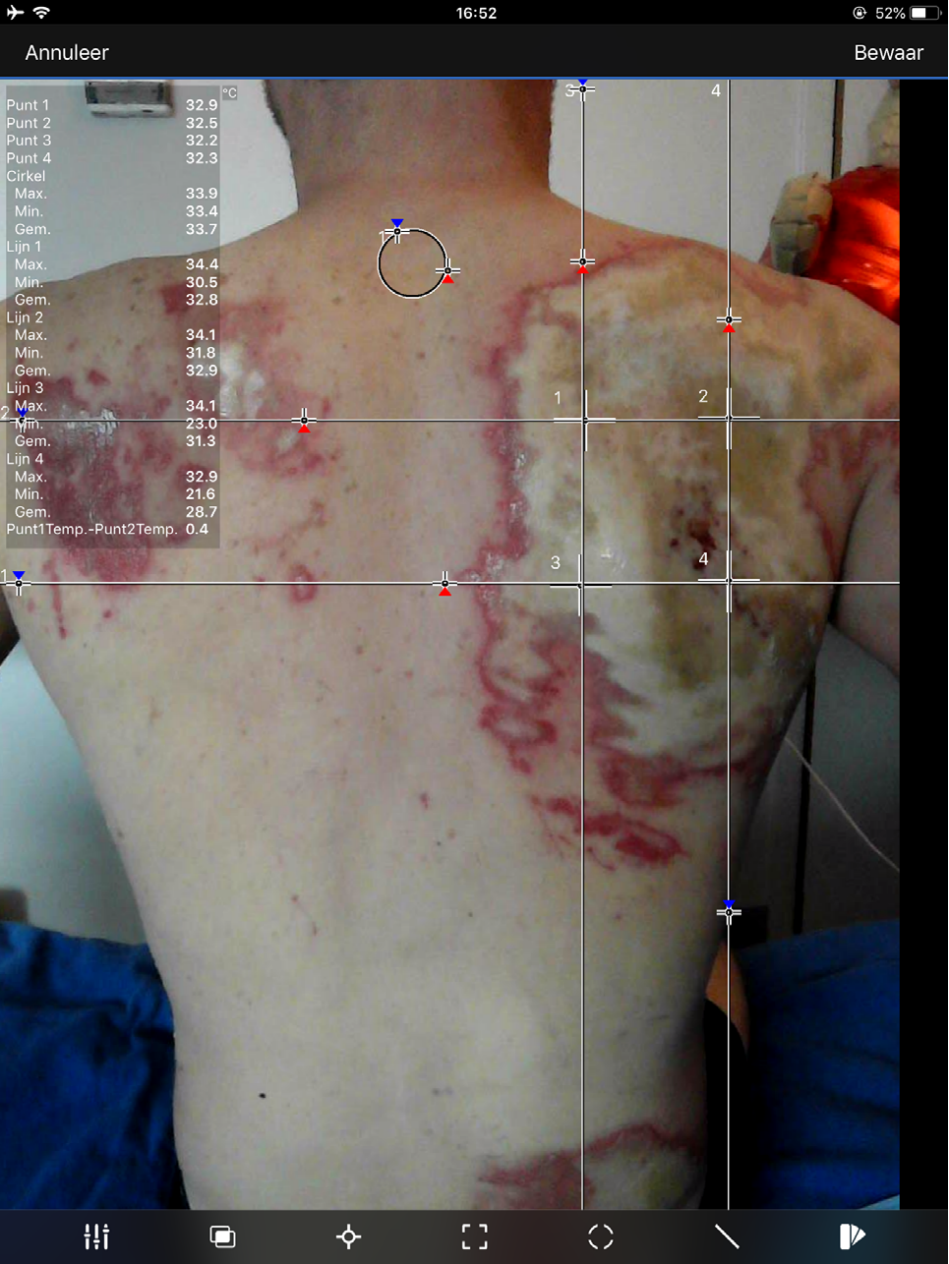
Analysis of a thermal image in the software application [Color figure can be viewed at https://wileyonlinelibrary.com]

**Figure 4:**
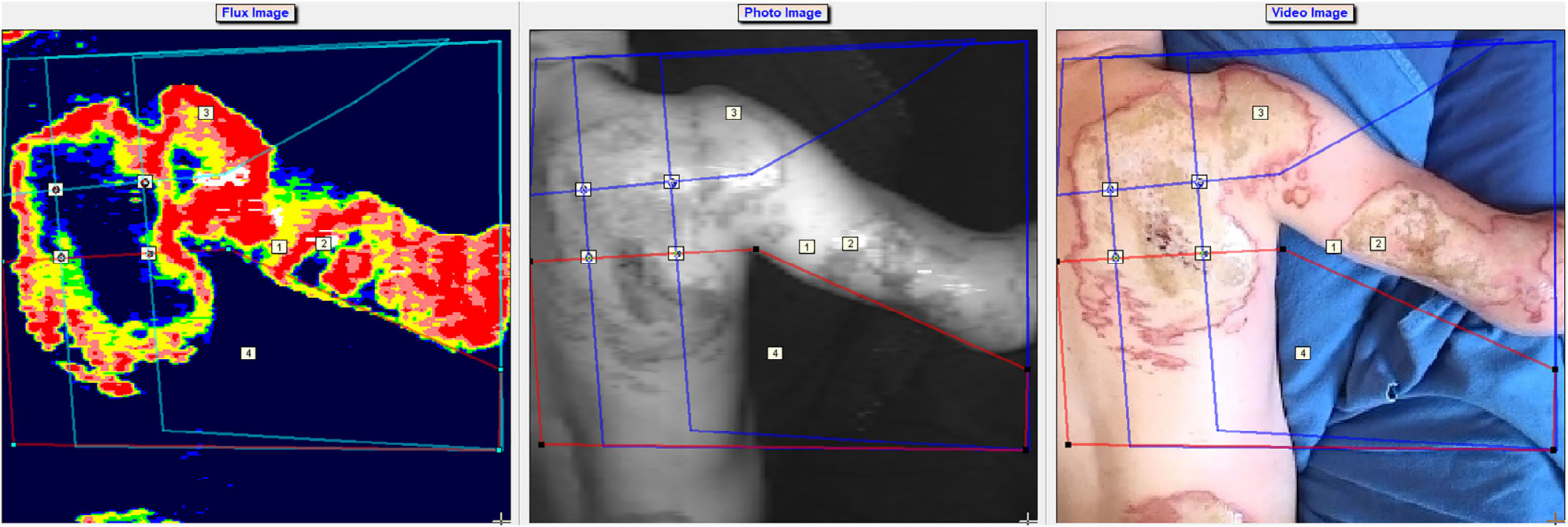
Analysis of a LDI scan in the corresponding software [Color figure can be viewed at https://wileyonlinelibrary.com]

Thermal images were analyzed using the corresponding software application on an iPad mini as shown in Figure 3. Depending on the size of the burn wound, one to five measurement points were chosen within the wound, following the principle of a standardized measurement algorithm, as described by Verhaegen et al.^26^ This was done in a systematic fashion by inserting horizontal and vertical lines based on anatomic landmarks on the normal VGA photo of the acquired thermal image. The points at which the lines crossed were assigned as measurement points. On the VGA picture, thermographic colors are not visible. Accordingly, bias in the selection of measurement points on the basis of thermographic information was prevented. In addition to the measurement points, a circle was outlined as the reference area (i.e., healthy skin) of which the mean temperature was calculated. The temperature difference between the measurement points and the reference area was calculated by one of the researchers (MC or LH) and expressed as Δ*T*. In the LDI software version V3.0 similar measurement points in the burn wound were analyzed by constructing the same lines as in the thermal image based on the chosen anatomic landmarks (Figure 4). LDI results were expressed in perfusion units (continues scale) and healing potential categories (ordinal scale). Only measurements consisting of more than 75% of red, yellow, or blue on the LDI image were included in the analysis. This was done to eliminate the effect of heterogeneous areas consisting of different healing potentials on the thermography results.

### 2.5 Statistical analysis

The correlation between Δ*T* values and perfusion units was expressed by the Pearson correlation coefficient (Pearson's *r*). A Pearson's *r* ≥ 0.7 was considered a strong positive correlation.^27^ Mean Δ*T* values were compared between the healing potential categories assessed by LDI using ANOVA analysis. To illustrate the ability of the thermal imager to discriminate between healing potential categories, receiver operating characteristic (ROC) curves were created. A ROC curve plots the true positive rate (sensitivity) on the *x*‐axis against the false positive rate (1‐specificity) on the *y*‐axis at various threshold settings (Δ*T* values). Two ROC curves were obtained, one for distinguishing between healing potential categories <14 and ≥14 days, and one for distinguishing between healing potential categories ≤21 and >21 days. The area under the curve (AUC) value of both ROC curves was calculated to express how well the thermal imager discriminates between the healing potential categories. An AUC value of 0.5 equals no discriminating ability, between 0.7 and 0.8 equals a fair discriminating ability, between 0.8 and 0.9 equals a good discriminating ability, and between 0.9 and 1 an excellent discriminating ability. For each ROC curve, one Δ*T* value was chosen with a high specificity. Data were analyzed using SPSS, Version 25.0 (IBM, Armonk, New York).

## 3 RESULTS

**Figure 5:**
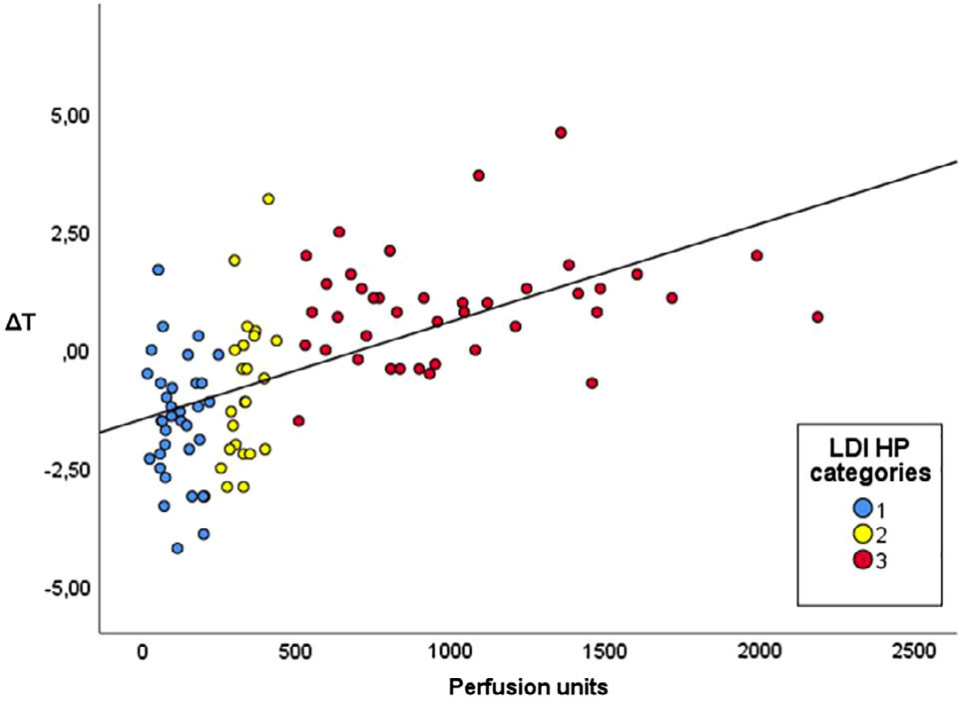
Scatterplot that illustrates the relationship between perfusion units obtained by LDI and the mean Δ*T* values obtained by thermography [Color figure can be viewed at https://wileyonlinelibrary.com]

**Figure 6:**
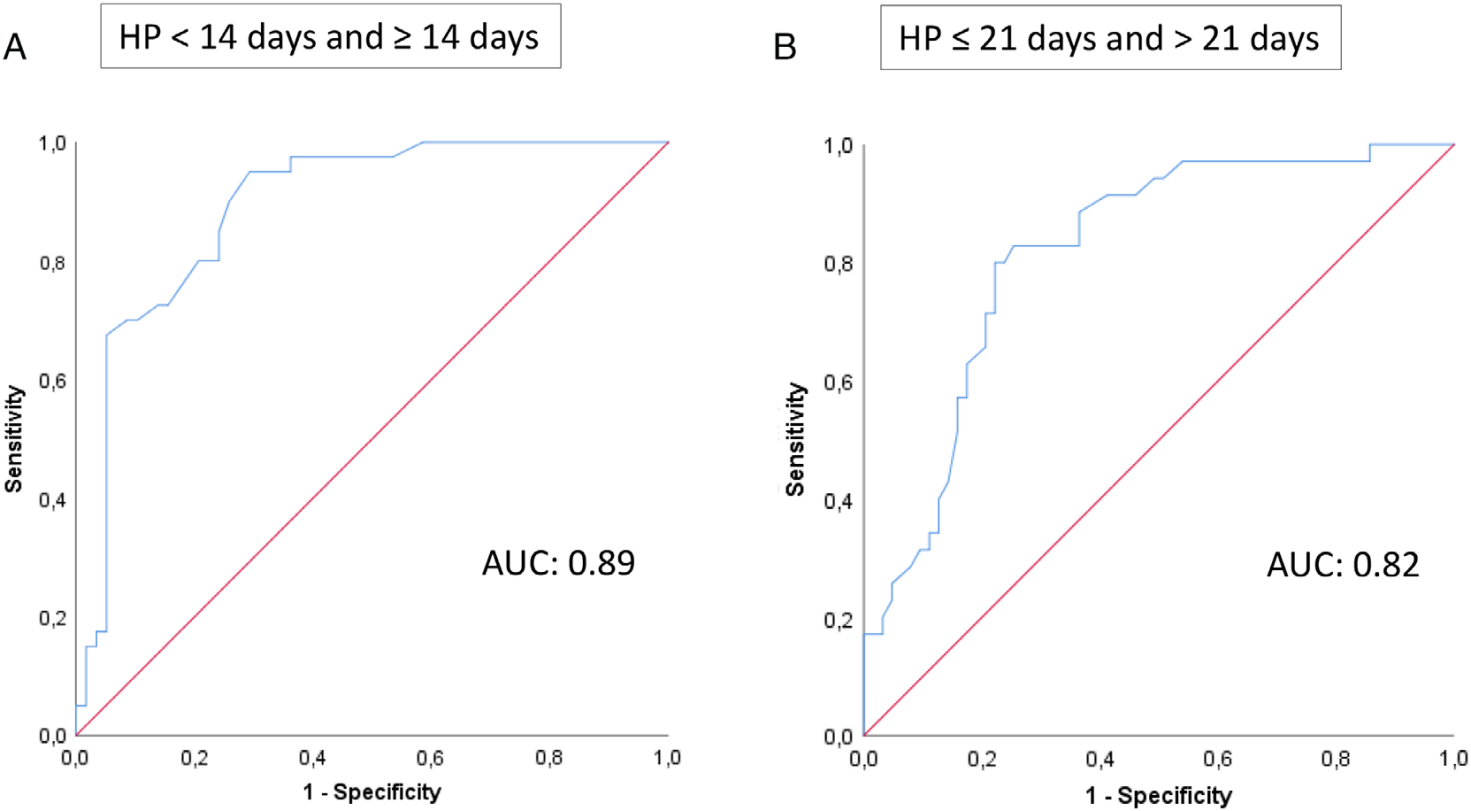
Two ROC curves that express how well the thermal imager can differentiate between healing potential categories <14 and ≥14 days (left), and healing potential categories ≤21 and >21 days (right). AUC, area under the curve [Color figure can be viewed at https://wileyonlinelibrary.com]

**Figure 7:**
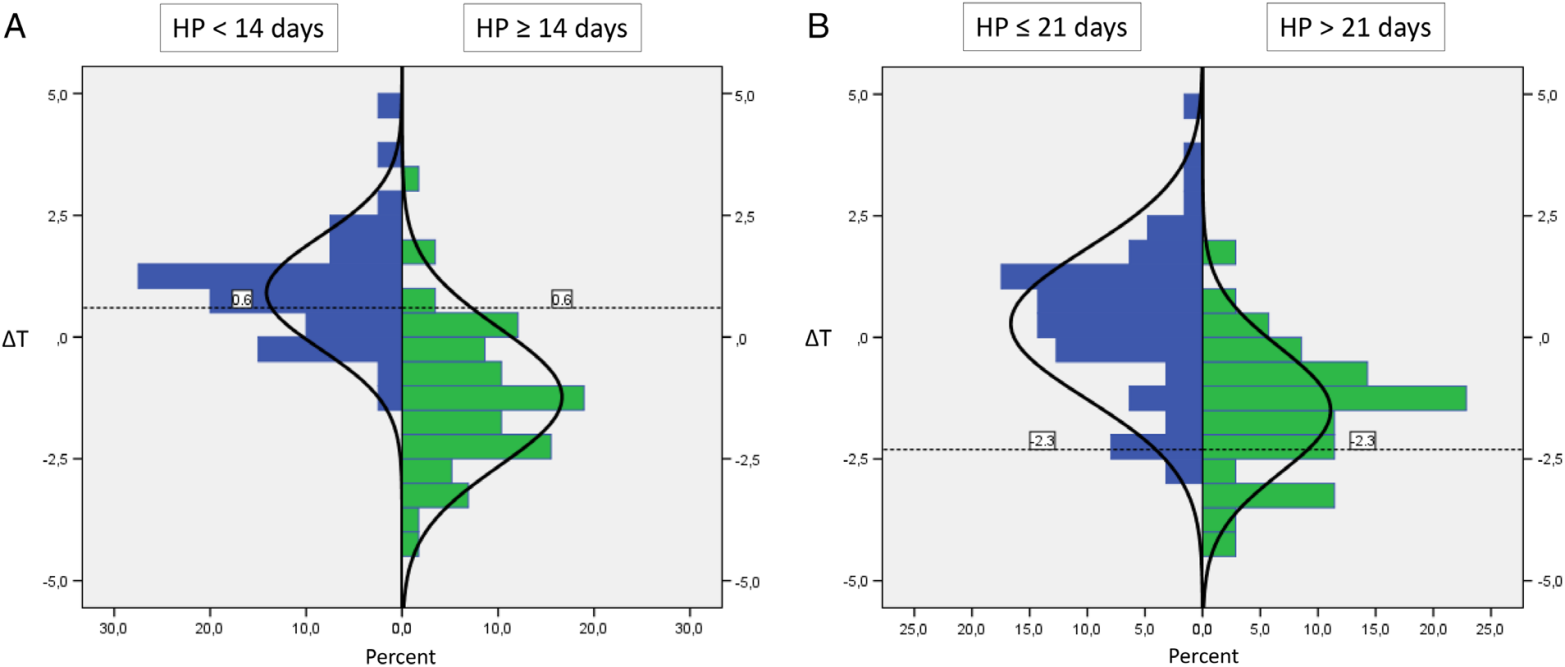
Two histograms illustrating the distribution of all burn wounds in percentages across Δ*T* values. Histogram (A) discriminates between burn wounds that heal in less than 14 days (left) and burn wounds that heal in a time period of 14 days and more (right). Histogram (B) discriminates between burn wounds that heal within 21 days (left), and in more than 21 days (right). The dotted lines in both histograms show the Δ*T* cutoff points of 0.6°C and −2.3°C, which were calculated based on the ROC analyses [Color figure can be viewed at https://wileyonlinelibrary.com]

Patient and burn wound characteristics are summarized in Table 1. A total of 4 3 burn wounds in 32 patients were included, of which 3 patients were < 18 years. Most of the participants were male (64.5%). The majority of included burn wounds were flame burns (52.5%). Burn wounds were most often located on legs (33%), arms (30%), or the trunk (21%). Mean Δ*T* values were significantly different (*P*‐value <.001) for each healing potential category, as shown in Table 2. The mean Δ*T* value for burn wounds with healing potential <14 days was higher than 0°C (0.91°C), whereas mean Δ*T* values for the other healing potential categories were below 0°C (14‐21 days: −0.81°C and >21 days: −1.50°C). In Figure 5, we plotted the Δ*T* values against perfusion units (assessed by LDI) for each of the healing potential categories. A moderate positive correlation between Δ*T* and mean perfusion units was found (Pearson's *r* = 0.6, *P* < .001). The ability of the thermal imager to distinguish between healing potential categories <14 and ≥14 days (Figure 6A) and ≤21 and >21 days (Figure 6B) is illustrated by two ROC curves, with an estimated AUC of 0.89 (95% CI 0.83‐0.96, *P*‐value <.001) and 0.82 (95% CI 0.73‐0.90, *P*‐value <.001), respectively. These AUCs both reflect a good ability to discriminate between the healing potential categories. Based on a sensitivity of 68% and a specificity of 95%, a Δ*T* cutoff value of 0.6°C was calculated to discriminate between burn wounds that heal within 14 days and burn wounds that take longer to heal. To discriminate between healing potential categories ≤21 and >21 days, a cutoff value of −2.3°C was calculated, associated with a sensitivity of 30% and a specificity of 95%. Figure 7 illustrates the distribution of burn wounds in percent across Δ*T* values, divided per healing potential category (assessed by LDI). The dotted lines show the established Δ*T* cutoff value. For example, Figure 7A shows burn wounds with a healing potential of <14 days on the left side of the diagram, and burn wounds with a healing potential of ≥14 days on the right side of the diagram. Burn wounds with a Δ*T* value higher than the cutoff value of 0.6°C are classified by thermography as healing <14 days (all burn wounds in the left and right upper quadrants). The sensitivity of 68% is calculated by dividing all the true positives (burn wounds in the left upper quadrant) by the true positives plus the false negatives (burn wounds in the left upper and left lower quadrants). The specificity of 95% is calculated by dividing all the true negatives (burn wounds in the right lower quadrant) by the true negatives plus the false positives (burn wounds in the right upper and right lower quadrants).

**Table 1:** Patient and burn wound characteristics

	Value, *N*	%
Burn wounds	43	
Patients	31	
Sex		
Male	20	64.5%
Female	11	35.5%
Age of patient, years		
Mean (SD)	40 (22)	
Assessment, postburn day		
Median (range)	3 (2‐5)	
TBSA, %		
Median (range)	6 (1‐28)	
Cause of burn		
Flame	16	52%
Oil	6	19%
Scald	5	16%
Contact	2	6.5%
Other	2	6.5%
Burn wound location	*N* = 43	
Trunk	9	21%
Arm	13	30%
Hand	5	11%
Leg	14	33%
Foot	2	5%

Abbreviations: SD, standard deviation; TBSA, total body surface area.

**Table 2:** Number of measurement points and mean Δ*T* value for each burn wound category, assessed by means of LDI

	HP <14 days	HP 14‐21 days	HP >21 days	*P*‐value
Measurement points, *N* (%)	40 (41%)	23 (23%)	35 (36%)	
Mean Δ*T*, °C (95% CI)	0.91 (0.054 to 1.28)	−0.82 (−1.48 to −0.15)	−1.50 (−1.94 to −1.06)	<.001^a^

Abbreviations: CI, confidence interval; HP, healing potential.

a Statistics. ANOVA analysis.

## 4 DISCUSSION

The potential predictive value of thermography in burn wound assessment was introduced in 1961.^28^ More than a decade later, the diagnostic technique was first tested on a large series of burn patients in a study by Hackett et al.^23^ This study demonstrated that it was more accurate than clinical evaluation (75% vs 90%). Many studies have been conducted since, using a wide range of thermal imagers, overall reporting promising results.^17^, ^18^, ^19^, ^20^, ^21^, ^22^ However, cumbersome and low‐resolution equipment hampered the regular use of thermography in clinical practice. Due to technological advancements, thermal imagers became smaller, faster, and more affordable. Recently, low‐cost, smart phone‐based thermal imagers have become available.

This study demonstrates a good validity of the thermal imager for the assessment of burn wound healing potential. Two Δ*T* cutoff values of 0.6 and −2.3°C were provided, which allow for discrimination between burn wounds that heal in <14 and ≥14 days, and for discrimination between burn wounds that heal in ≤21 and >21 days, with corresponding sensitivity values of 68% and 30%, respectively, and specificity values of 95% for both. Optimal cutoff values can vary for different test purpose, depending on the desired sensitivity and specificity values. This is visualized in Figure 7. By changing the cutoff value (dotted line), the amount of burn wounds that are categorized in each of the healing potential categories also change, which consequently leads to other sensitivity and specificity values. In this study, we have selected two Δ*T* cutoff values that are accompanied by a high specificity rather than a high sensitivity. As a result, few burn wounds will be classified in healing potential categories <14 and >21 days due to a lower sensitivity, but of all the wounds that are identified in these categories, 95% is correctly classified. This is important not only to confidently provide conservative treatment to burn wounds that are predicted to heal in a time period of less than 14 days, but also to avoid the possibility of performing unnecessary surgery on burn wounds that would have healed spontaneously.

There are two challenges relating to the use of thermography, which may have negatively influenced our results. First, selecting the most appropriate reference area of unaffected skin is a critical part of the thermography analysis as it greatly affects the resulting Δ*T* value. This task is particularly challenging in patients with burn wounds located on extremities. In this situation, the reference area can be chosen either next to the burn wound or on the contralateral extremity. The latter option is supported by the hypothesis that identical locations on both extremities should have the same body temperature.^29^ However, we found substantial differences in temperature between extremities. Possible causes are temperature rising due to dressings, garments, or spreading inflammation on the affected side, as well as warmth caused by the administration of intravenous fluids on the unaffected side. In addition, positioning of limbs and patient‐specific variability may also have an effect on the measured skin temperature. For these reasons, we decided to use a reference area without visible redness next to the burn, with a distance of at least 3 cm from the burn. Nonetheless, there is a possibility that edema and some inflammation might have led to a higher temperature in the reference areas, causing a larger Δ*T* value. Second, environmental influences, such as wound exposure time, evaporation, and humidity, for which we were not able to control completely, may have had an effect on the measured skin temperature as well.

In this study, a significant Pearson's correlation coefficient of 0.6 (*P* < .001) was calculated, indicating a positive, moderate correlation between mean Δ*T* values and mean perfusion units (LDI). This finding falls within the range of the results of other studies, reporting Pearson's correlation coefficients of 0.50 (*P* = <.01) and 0.73 (*P* = <.01).^18^, ^20^ The Δ*T* cutoff values that were selected in this study differ from cutoff values selected in other studies.^22^, ^30^ The reason for this difference is that we based our cutoff value on the preferred specificity, whereas in other studies cutoff values were selected with both the highest sensitivity and specificity.

The results of this study are in line with the results obtained with the previously studied thermal imager, in terms of Δ*T* values and corresponding sensitivity and specificity. For example, the current study shows a specificity of 100% and sensitivity of 17% at a cutoff value of −3.0°C (data not shown), whereas the previous study showed a specificity of 100% and sensitivity of 13% at a cutoff value of −3.2°C.^25^ Although these two studies show the same trend, the overall validity (i.e., AUC and sensitivity/specificity values) is higher in the current study. This difference may be explained by the improved software, along with the higher resolution visual VGA imager that is built in. Another, more likely, explanation may be found in the study method. In this study, we included carefully outlined measurement points, and excluded measurements that contained heterogeneous healing potential areas, whereas in the previous study a relatively large area was assessed within the burn wound. Furthermore, the observed healing time (i.e., >95% epithelialization) was chosen as a reference standard in the previous study, as well as in other clinical studies.^18^, ^20^, ^30^ We believe this reference standard has several limitations. First, it is challenging to assess the actual healing day of patients who are discharged from the burn unit, as patients are unlikely to visit the outpatient clinic on the same day as 95% epithelialization has occurred, and patients generally do not have the capacity to assess this. Second, burn physicians often decide on relatively early surgical treatment when they expect a healing potential over 21 days with the aim to minimize problematic scarring. Consequently, the actual healing time of these wounds cannot be assessed, which might have led to an underrepresentation of burn wounds with a healing potential between 14 and 21 days in those studies.

The decision to use LDI as a reference standard in the current study was based on a recent systematic review that investigated the measurement properties (i.e., reliability, validity) of all techniques that aim to assess burn wound depth or healing potential and concluded that LDI is the most favorable technique.^31^ Besides the measurement properties, however, feasibility is an important aspect that needs to be evaluated prior to choosing an instrument.^32^ In terms of feasibility, it must be noted that LDI has several disadvantages: it can only be used after 2 days postburn, patients must lie still during measurements, and the device is extremely expensive and cumbersome to carry around.^13^, ^33^ These practical limitations do not apply to the thermal imager, as it is an affordable, easily accessible imager, which provides easy and fast measurements (2 seconds) and analyses (2 minutes). Moreover, previous research suggested that thermography may perform optimal within the first 3 days postburn, as wound granulation might influence the accuracy of measurements,^17^ whereas LDI is advised to use after 48 hours postburn.

As the thermal imager provides obvious advantages in terms of feasibility and accessibility, we consider it a promising technique to be used for two different purposes. First, as a triage instrument in local hospitals and general practices. In this situation, it is most important to discriminate between burn wound healing potential categories <14 and ≥14 days. Using the 0.6°C cutoff value, physicians can distinguish between burn wounds that can stay in nonspecialized centers for conservative treatment (Δ*T* higher than 0.6°C), and burn wounds that need to be referred to a burn center for further diagnosis and treatment (Δ*T* lower than 0.6°C). Second, the thermal imager may play an important diagnostic role in burn centers. In this case, both cutoff values (0.6 and −2.3°C) are equally useful. Burn wounds with a Δ*T* value higher than 0.6°C can be discharged from the burn center sooner and referred to the outpatient clinic for conservative treatment and follow‐up. Furthermore, burn wounds with a Δ*T* value below −2.3°C are identified as having a healing potential of >21 days, which can benefit from early surgical treatment. The quite large “intermediate” group of burn wounds with Δ*T* values between these two cutoff values needs to be monitored and evaluated further. We then advise to perform an additional LDI when available.^31^ The important advantage of using the thermal imager in a burn center is that fewer patients would be exposed to the time‐consuming and expensive process of LDI scanning. We believe this may have a positive impact on patient distress as well as the efficiency of clinical staff. Before the thermal imager can be implemented in clinical practice, future research is required to evaluate its validity for determining burn wound healing potential on day 1 and 2 postburn, and its use as an add‐on test to clinical evaluation. In this future study, it would be interesting to record additional local and systemic factors which might influence wound healing, and to collect a larger sample size to compare the performance of thermography on different locations of the body. Furthermore, we would prolong the follow‐up period so that the final scar quality can be assessed.

## 5 CONCLUSION

This study demonstrated a good validity of the thermal imager for the assessment of burn wound healing potential, using LDI as a reference standard. In addition, two cutoff values were established to discriminate between burn wounds that heal in more or less than 14 days, and in more or less than 21 days. The handheld thermal imager is easily accessible, affordable, and feasible. Ultimately, we consider it a promising technique to be used for triage in local hospitals and general practices, and as a valuable addition to clinical evaluation in burn centers.

## CONFLICT OF INTEREST

None of the authors has a financial interest in the devices mentioned in this study.
